# Potential for Computational Genotoxicity: A Report on Symposium 3 of the 53rd Annual Meeting of the Japanese Environmental Mutagen and Genome Society (JEMS), 2024

**DOI:** 10.1186/s41021-026-00358-y

**Published:** 2026-03-12

**Authors:** Naoki Koyama, Ayako Furuhama, Naomi L. Kruhlak, Nicolas Ken Shinada, Kazuki Izawa

**Affiliations:** 1 Safety and Bioscience Research Department, Translational Research Division, Chugai Pharmaceutical Co., Ltd, 216 Totsuka-cho, Totsuka-ku, Yokohama, Kanagawa 244-8602 Japan; 2 Division of Genome Safety Science, National Institute of Health Sciences (NIHS), 3-25-26 Tonomachi, Kawasaki-ku, Kawasaki City, Kanagawa 210-9501 Japan; 3 Center for Drug Evaluation and Research, US Food and Drug Administration, 10903 New Hampshire Avenue, Silver Spring, MD 20993-0002 USA; 4 SBX Corporation, 5 Chome-10-25 Higashi Gotanda, Shinagawa City, Tokyo 141- 0022 Japan

**Keywords:** (Q)SAR, ecNGS, Computational genotoxicity, CPCA, AI, Regulatory application, Genotoxicity prediction, Domain knowledge, Carcinogenesis, Hazard identification

## Abstract

Symposium 3 of the 53rd Annual Meeting of the Japanese Environmental Mutagen and Genome Society (JEMS), entitled “Potential for Computational Genotoxicity,” was held at Shujitsu University, Okayama, Japan, on December 8, 2024. The symposium discussed the application of advanced informatics technologies, such as (quantitative) structure-activity relationship ((Q)SAR) and error-corrected next-generation sequencing (ecNGS), to the field of genotoxicity within the framework of computational genotoxicity. In this symposium, we invited three scientists who are global leaders in the field of computational genotoxicity. This report summarizes the key discussions and presentations from the symposium. The organizers hope this summary will increase awareness of computational genotoxicity.

## Background

The 53rd Annual Meeting of the Japanese Environmental Mutagen and Genome Society (JEMS) was held on December 7 and 8, 2024 in Okayama, Japan, with the theme “Saving the Environment & Genome in the Future.” In recent years, genotoxicity studies have increasingly emphasized the use of (quantitative) structure-activity relationship ((Q)SAR) modeling for predicting Ames mutagenicity [1, 2] and error-corrected next-generation sequencing (ecNGS) for detecting low-frequency gene mutations based on the principles of double-strand consensus sequencing [3–10]. Symposium 3 brought together experts from cheminformatics and bioinformatics to discuss the latest advances in informatics and ecNGS within the framework of computational genotoxicity and their regulatory applications. The symposium aimed to create a computational genotoxicity platform for a more data-informed approach to genotoxicity assessment for drug products and various environmental mutagens. The symposium program was as follows:

- Potential for Computational Genotoxicity (Introduction), Naoki KOYAMA, Safety and Bioscience Research Dept. Translational Research Division, Chugai Pharmaceutical Co., Ltd.
- US FDA Experience in the Regulatory Application of (Q)SAR, Naomi Louise KRUHLAK, US Food and Drug Administration, Center for Drug Evaluation and Research.
- Toward Fully Automated Genotoxicity Prediction, Nicolas Ken SHINADA, SBX Corporation.
- Importance of Domain Knowledge in Data Analysis: ecNGS Analysis, Kazuki IZAWA, Division of Genome Safety Science, National Institute of Health Sciences.

Here, we summarize the symposium presentations and discuss future perspectives for research in computational toxicology.

### Opening Address

#### Naoki KOYAMA (Chugai Pharmaceutical Co., Ltd.)

Dr. Naoki Koyama, the symposium chair, opened the event and introduced the basic concepts of computational genotoxicity. He first distinguished between “computational toxicology” and “computational genotoxicity”. While computational toxicology is a recognized field concerned with using computer-based models to predict the interactions of biological organisms (at population, individual, cellular, and molecular levels) with chemical agents [11], “computational genotoxicity” has not yet been formally defined. Dr. Koyama described computational genotoxicity as a field that integrates a wide variety of informatics data to elucidate, analyze, and predict genotoxicity-related phenomena from multiple perspectives. He emphasized how advanced informatics technologies, such as (Q)SAR and ecNGS, can be applied within this framework and adapted to the field of genotoxicity.

### US FDA Experience in the Regulatory Application of (Q)SAR

Naomi Louise KRUHLAK, US Food and Drug Administration, Center for Drug Evaluation and Research.

Dr. Naomi Kruhlak, a pioneer of (Q)SAR research in genetic toxicology, delivered a comprehensive presentation on the methodology and its practical application in the regulation of pharmaceuticals. She explained that (Q)SAR computational models can predict toxicity based solely on a chemical’s structure. The U.S. Food and Drug Administration’s Center for Drug Evaluation and Research (FDA/CDER) uses (Q)SAR models to provide predictions for chemicals under review, such as drug impurities, when robust experimental data are unavailable [12, 13]. Dr. Kruhlak highlighted that the International Council for Harmonisation (ICH) of Technical Requirements for Pharmaceuticals for Human Use M7 guideline, first published in 2014, recommends (Q)SAR approaches as alternatives to the Ames test for evaluating the mutagenicity of impurities [14]. This has led to a growing interest in (Q)SAR modeling for drug development and regulation [12, 13]. Dr. Kruhlak also presented her most recent work in modeling the carcinogenic potency of a sub-class of mutagenic impurities―nitrosamines―resulting in the regulatory implementation of a categorical SAR model to determine acceptable intake limits [15, 16]. In closing, she shared practical considerations for the regulatory use of (Q)SAR models to promote transparency and enhance predictive performance [17, 18].

### Toward Fully Automated Genotoxicity Prediction

#### Nicolas Ken SHINADA (SBX Corporation)

Dr. Nicolas Ken Shinada addressed the limitations of traditional genotoxicity assessment methods, such as the Ames test, which are expensive, time-consuming, and labor-intensive. He emphasized that reliable in silico methods, i.e., artificial intelligence (AI) tools, are fundamental to the drug design process. Dr. Shinada presented his recent research utilizing machine learning (ML) and natural language processing (NLP) to develop cost-effective and scalable in silico mutagenicity prediction models [19]. However, constructing reliable models for genotoxicity remains challenging due to the need for substantial training data. While structured resources such as the Hansen mutagenicity benchmark dataset [20] and (Q)SAR databases offer some data for Ames mutagenicity, a vast amount of valuable information embedded in scientific literature remains underutilized. This is primarily due to the complexity of extracting structured data from unstructured textual sources. He presented a novel text-mining framework using a transformer-based model, MutaPredBERT [21], which reformulates the task as a biomedical question-answering problem to extract mutagenic information directly from scientific abstracts. The model achieved a high macro F1-score of 0.88 on a curated dataset, demonstrating its accuracy. Additionally, Dr. Shinada highlighted a 2022 ML study on optimizing molecular fingerprint-based models through systematic feature selection, in which algorithms like descriptors aggregation and recursive feature elimination significantly improved prediction performance [19]. He emphasized the importance of a multidisciplinary strategy combining cheminformatics and NLP to modernize mutagenicity assessment.

### Importance of Domain Knowledge in Data Analysis: ecNGS Analysis

Kazuki IZAWA (Division of Genome Safety Science, National Institute of Health Sciences).

Dr. Kazuki Izawa gave a presentation on ecNGS-based data analysis. He focused on ecNGS, an advanced informatics technology capable of detecting genome-wide low-frequency mutations with double-strand consensus sequencing. Dr. Izawa emphasized that domain knowledge is crucial for developing ecNGS technologies. This includes a deep understanding of chemical mutagen, genome structure, mutation types, and potential artifacts in the data analysis pipeline. Specifically, he explained that the DNA synthesizing steps in library preparation, such as end-repair, make artifacts that result in residual errors in low-frequency mutation detection. He demonstrated that identifying and filtering these artifacts is essential for accurate mutation quantification. Dr. Izawa also introduced research analyzing genome-wide mutation profiles associated with specific chemical mutagens, which provides a broader view of genomic effects than single-locus analysis [22]. His presentation highlighted the critical synergy between data-driven science and human expertise, emphasizing the need for genotoxicity researchers to collaborate with informatics scientists to fully harness the potential of these technologies.

## Conclusion

The 53rd Annual Meeting of JEMS in 2024 was a significant success, attracting over 200 participants. Symposium 3 effectively brought together key stakeholders and researchers from various sectors to discuss the current state of computational genotoxicity. Participants gained an understanding of computational genotoxicity, a new and rapidly evolving field. The symposium showed how informatics technologies like (Q)SAR and ecNGS are revolutionizing genotoxicity prediction. Dr. Koyama introduced the foundational concepts, while Dr. Kruhlak shared practical regulatory experience with (Q)SAR. Dr. Shinada presented cutting-edge research on ML prediction models, and Dr. Izawa highlighted the indispensable role of domain knowledge in interpreting complex ecNGS data. The symposium demonstrated that computational genotoxicity is an emerging interdisciplinary field that integrates informatics and biological expertise to enable more efficient and accurate genotoxicity evaluation.

The symposium organizers, Dr. Naoki Koyama and Dr. Ayako Furuhama, extend their sincere thanks to Professor Masahiko Watanabe, the President of the 53rd Annual Meeting of JEMS, the organizing committee, and the staff of Senkyo Co., Ltd., for their support and contributions to the symposium’s success. As the organizers, we extend our sincere gratitude to all who participated in this symposium.

## Data Availability

No datasets were generated or analysed during the current study.

## Declarations

### FDA disclaimer

This manuscript reflects the views of the authors and should not be construed to represent FDA’s views or policies.

## Abbreviations

AI: Artificial intelligence
CPCA: Carcinogenic Potency Categorization Approach
ecNGS: Error-corrected next-generation sequencing
ICH: International Council for Harmonisation of Technical Requirements for Pharmaceuticals for Human Use
JEMS: The Japanese Environmental Mutagen and Genome Society
(Q)SAR: (Quantitative) structure-activity relationship
ML: Machine learning
NLP: Natural language processing
